# Analysis of DNA relaxation and cleavage activities of recombinant *Mycobacterium tuberculosis *DNA topoisomerase I from a new expression and purification protocol

**DOI:** 10.1186/1471-2091-10-18

**Published:** 2009-06-11

**Authors:** Thirunavukkarasu Annamalai, Neil Dani, Bokun Cheng, Yuk-Ching Tse-Dinh

**Affiliations:** 1 Department of Biochemistry and Molecular Biology, New York Medical College, Valhalla, New York, USA

## Abstract

**Background:**

*Mycobacterium tuberculosis *DNA topoisomerase I is an attractive target for discovery of novel TB drugs that act by enhancing the accumulation of the topoisomerase-DNA cleavage product. It shares a common transesterification domain with other type IA DNA topoisomerases. There is, however, no homology between the C-terminal DNA binding domains of *Escherichia coli *and *M. tuberculosis *DNA topoisomerase I proteins.

**Results:**

A new protocol for expression and purification of recombinant *M. tuberculosis *DNA topoisomerase I (MtTOP) has been developed to produce enzyme of much higher specific activity than previously characterized recombinant enzyme. MtTOP was found to be less efficient than *E. coli *DNA topoisomerase I (EcTOP) in removal of remaining negative supercoils from partially relaxed DNA. DNA cleavage by MtTOP was characterized for the first time. Comparison of DNA cleavage site selectivity with EcTOP showed differences in cleavage site preferences, but the preferred sites of both enzymes have a C nucleotide in the -4 position.

**Conclusion:**

Recombinant *M. tuberculosis *DNA topoisomerase I can be expressed as a soluble protein and purified in high yield from *E. coli *host with a new protocol. Analysis of DNA cleavage with *M. tuberculosis *DNA substrate showed that the preferred DNA cleavage sites have a C nucleotide in the -4 position.

## Background

DNA topoisomerases are ubiquitous enzymes involved in the regulation of DNA supercoiling and overcoming topological barriers during replication, transcription, recombination and repair. In bacteria, the major classes of topoisomerases, type IA and type IIA, modify DNA topology by transiently cleaving and rejoining one or two strands of DNA respectively [1,2]. Both of these classes form a 5'-phosphotyrosyl enzyme-DNA linkage during the catalytic cycle of DNA cleavage and religation [1]. Topoisomerases are attractive targets for development of new anti-infectives [3]. Bacterial DNA gyrase and topoisomerase IV from the type IIA class are targets of antibiotics such as quinolones and fluoroquinolones. These antibiotics exhibit their bactericidal properties by trapping the covalent protein-DNA complexes formed by DNA gyrase or topoisomerase IV [4,5]. Although fluoroquinolones are effective against a broad spectrum of bacteria, alarming increase in fluoroquinolone-resistant pathogens warrants the need to develop novel drugs against new cellular targets.

Bacterial topoisomerase I, responsible for relaxing negatively supercoiled DNA, is the most common type IA topoisomerase present in almost all bacteria [6,7]. *Escherichia coli *topoisomerase I (EcTOP) is the well studied prototype for type IA topoisomerase [8]. EcTOP relaxes negatively supercoiled DNA through a magnesium-dependent, ATP-independent catalytic mechanism. No specific inhibitor for bacterial topoisomerase I, effective at a relevant clinical and physiological concentration, has been identified. Bacterial topoisomerase I, by virtue of its presence in nearly all bacterial genomes, and in view of its association with DNA during the vulnerable stage of cleavage-religation, could be utilized as a target for novel antimicrobials [3]. This strategy could be useful in developing drugs to treat highly fatal bacterial diseases like tuberculosis [9]. The fact that approximately one-third of the world's population is affected by tuberculosis indicates the need to develop effective drugs against this disease [10]. Also, since multiple drug resistance is common in *Mycobacterium tuberculosis*, it would be significant if a novel antibiotic targeting *M. tuberculosis *DNA topoisomerase I can be developed [9].

A logical first step towards finding inhibitors selective to *M. tuberculosis *topoisomerase I is to characterize the DNA modification ability of this enzyme. In this study, we describe a new expression and purification protocol for recombinant *M. tuberculosis *topoisomerase I capable of producing milligrams of pure protein. We also report the first detailed characterization of this enzyme with respect to its DNA cleavage sites and relaxation activity under different assay conditions.

## Results

### Expression and purification of *M. tuberculosis *topoisomerase I

Genome sequencing of *M. tuberculosis *H37Rv strain has revealed the presence of *topA *gene Rv3646c which encodes a DNA topoisomerase I (MtTOP) comprising of 934 amino acids with an estimated molecular weight of 102.3 kDa [11]. Previously, Yang et al [12] have cloned and purified DNA topoisomerase I from *M. tuberculosis *Erdman strain in *E. coli *BL21 (DE3). Our efforts to express and purify recombinant MtTOP in *E. coli *BL21 (DE3) similarly by induction of the T7 promoter were frustrated by the insolubility of the expressed protein. Difficulties have also been encountered by other researchers [13] while trying to use recombinant DNA topoisomerase I gene present in genomic libraries of *M. tuberculosis *and *Mycobacterium smegmatis *to complement the temperature dependent deficiency of topoisomerase I (*topA*) function in *E. coli *strain AS17 [14]. Difference in codon usage was surmised to be one of the possible reasons behind this result [13]. We overcame these difficulties by expressing MtTOP from a recombinant plasmid pLIC-MTOP in an *E. coli *Arctic express (DE3)RP strain (Stratgene) at low temperatures (12°C). The Arctic express (DE3)RP strain contained a chromosomally integrated T7 RNA polymerase which was expressed from the *lacUV5 *promoter. Induction of T7 RNA polymerase protein synthesis with IPTG resulted in the expression of the T7 promoter-driven recombinant protein. In addition, the Arctic express (DE3)RP strain expressed cold chaperonin proteins (Cpn10 and CPn60) and extra copies of tRNAs (recognizing arginine and proline codons) that facilitated the expression of recombinant proteins by overcoming issues of protein solubility and codon bias respectively.

**Figure 1:**
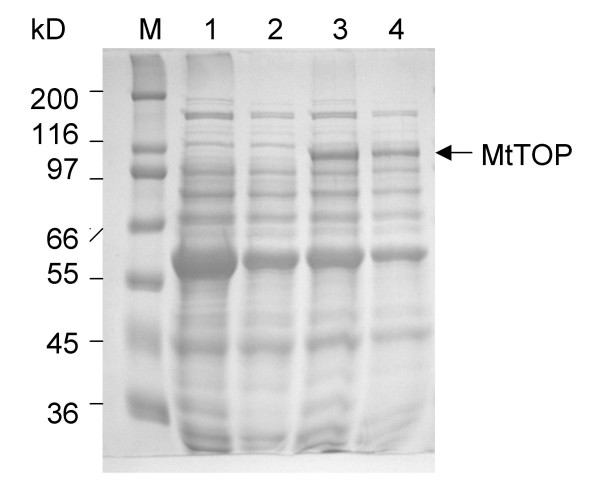
**Expression of recombinant MtTOP in *E. coli *Arctic express (DE3)RP strain**. SDS PAGE analysis of total cell lysate (lanes 1,3) and soluble cell lysate (lane 2,4) of Arctic express (DE3)RP cells transformed with pLIC-MTOP and induced with 0 mM (lanes 1,2) and 1 mM IPTG (lanes 3,4) at the end of 24 hours of induction in LB at 12°C. M: molecular weight standards.

**Figure 2:**
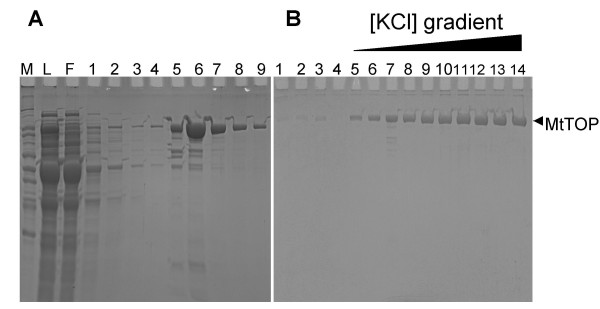
**SDS PAGE at different stages of MtTOP purification**. A. Wash (lanes 1–4) and elution (lanes 5–9) fractions of fusion protein passed through a Ni-NTA agarose column. Lanes M represents molecular marker and lanes L and F represent the initial load and flow through of the Ni-NTA agarose column before wash and elution. B. Wash fractions (lanes 1–4) and potassium chloride gradient elution fractions (lanes 5–14) of TEV digested fusion protein loaded onto single-strand DNA cellulose column.

Recombinant MtTOP was soluble and initially expressed as a hexa-histidine fusion protein only in the presence of IPTG (Figure 1). Purification of the fusion protein was achieved using nickel affinity chromatography. Subsequent SDS-PAGE analysis (Figure 2A) showed the predominant presence of only the fusion protein with the expected molecular weight. The hexa-histidine fusion tag was cleaved off by TEV protease treatment and MtTOP of high purity was eluted by increasing the potassium chloride gradient from a single-stranded DNA cellulose column (Figure 2B) [15]. The eluted fractions were pooled and dialyzed into storage buffer. Approximately 12 milligrams of purified protein was obtained from 7 L of bacterial culture in LB medium.

### Characterization of DNA relaxation activity of *M. tuberculosis *topoisomerase I

**Figure 3:**
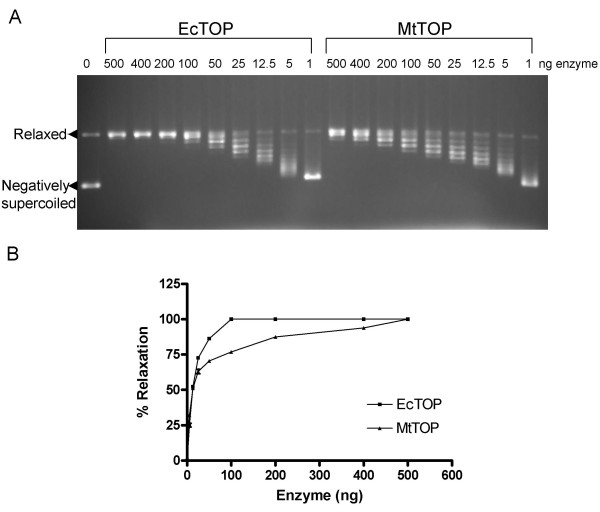
**Relaxation of negatively supercoiled DNA to determine unit activity**. A. 500 ng of negatively supercoiled plasmid DNA was subjected to relaxation as per the conditions in "materials and methods" with the indicated amount of EcTOP and MtTOP. B. DNA relaxation by different amounts of EcTOP and MtTOP was quantitated as percent relaxation for comparison. The percent relaxation was determined by dividing the distance between the negatively supercoiled band (SC); and the weighted center of the partially relaxed band (PR); by the distance between the supercoiled band (SC); and the fully relaxed band (FR). In simple terms, percent relaxation = (SC-PR)/(SC-FR) [18]. The percent relaxation values reported are averages of at least three independent experiments. Error bars denote the standard error of mean.

DNA relaxation assay was used to characterize the purified MtTOP. We compared the ability of MtTOP with that of similarly purified *E.coli *topoisomerase I (EcTOP) [16] in relaxing negatively supercoiled DNA by agarose gel electrophoresis. Initial assays evaluated the minimum amount of enzyme (MtTOP or EcTOP) required to bring about complete relaxation of negatively supercoiled DNA under standard conditions (Figure 3). One unit of enzyme was defined as the amount of enzyme required to relax 0.5 μg of negatively supercoiled plasmid DNA in 30 min at 37°C. Results indicated that 100 ng of EcTOP and 500 ng of MtTOP (Figure 3A) constitute one unit of enzyme activity. However, at lower concentrations of enzyme, = 12.5 ng, there is no difference between the ability of MtTOP and EcTOP in removing the negative supercoils from the plasmid DNA substrate (Figure 3).

**Figure 4:**
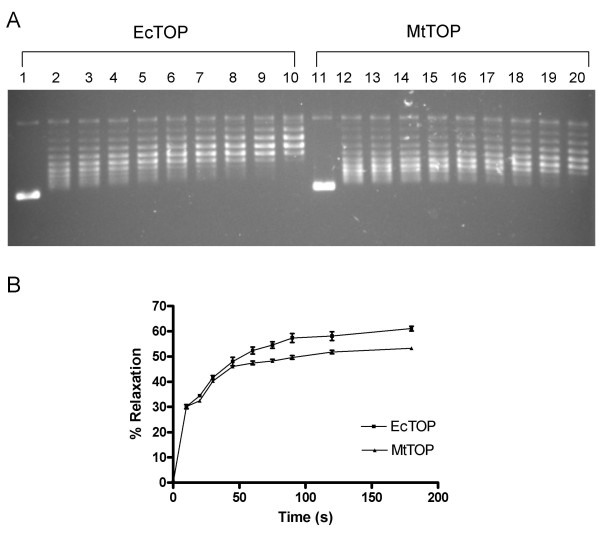
**Time course of DNA relaxation**. A. 50 ng of EcTOP (lanes 1–10) and MtTOP (lanes 11–20) was utilized in a relaxation assay as described in the "materials and methods" section over time course of 0, 10, 20, 30, 45, 60, 75, 90, 120, 180 sec respectively for each enzyme. B. Quantitation of the relaxation time course. The percent relaxation values reported are averages of at least three independent experiments. Error bars denote the standard error of mean.

For a more detailed analysis of the relaxation activity of the purified enzymes, a time course assay with 50 ng each of MtTOP and EcTOP was performed (Figure 4). At the early time points, the rate of removal of the negative supercoils by the two enzymes was similar. However, as the plasmid DNA substrate became partially relaxed, the relaxation activity of MtTOP was less efficient than EcTOP in removing the residual negative supercoils.

**Figure 5:**
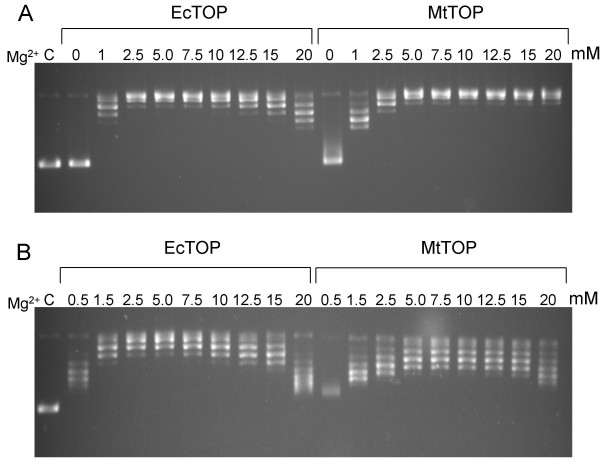
**Effect of Mg^2+ ^ion concentration on DNA relaxation**. Effect of different concentrations of Mg^2+ ^ion concentration ranging from 0–20 mM in DNA relaxation assays containing (A) high level, 1 enzyme unit corresponding to 100 ng of EcTOP and 500 ng of MtTOP; or (B) low level (50 ng) of EcTOP or MtTOP. C: control with no enzyme.

It has been a well known fact that Mg^2+ ^ions are required for the relaxation activity of bacterial type IA topoisomerases, including *E. coli *topoisomerase I [17,18]. We compared the Mg^2+^dependence of the relaxation activity of EcTOP and MtTOP using two different enzyme concentrations (50 ng or 1 unit in a 20-μl assay) and a range of Mg^2+ ^levels (Figure 5). At a lower enzyme concentration (50 ng), relaxation by EcTOP had a optimal range of Mg^2+ ^concentrations between 2.5 to 7.5 mM while the optimal range of Mg^2+ ^concentrations for MtTOP was slightly higher (5–12.5 mM) (Figure 5B). Similar optimal levels of Mg^2+ ^were found for the relaxation activities of both the EcTOP and MtTOP at higher enzyme concentrations equivalent to one unit of enzyme activity, with no relaxation observed in the absence of Mg^2+ ^(Figure 5A). The optimal Mg^2+ ^concentrations found here for MtTOP are higher than the 1 mM concentration determined in previous work [12]. In other studies involving the characterization of the topoisomerase I from *M. smegmatis*, the optimal Mg^2+ ^concentration for relaxation activity was found to be about 5 mM [19].

### Mapping of DNA cleavage sites using single-stranded DNA substrates

**Figure 6:**
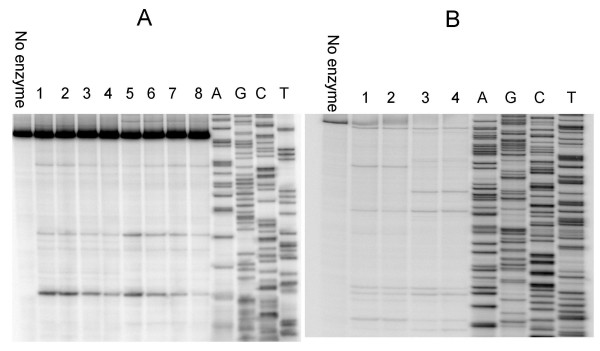
**Mapping cleavage sites on single-stranded DNA substrate**. A. Single-stranded DNA substrate Mtop (216 bases) amplified from *topA *gene of *M. tuberculosis *was utilized to map cleavage sites of EcTOP and MtTOP. Lanes 1–4: cleavage reactions containing 400, 300, 200, 100 ng of EcTOP respectively. Similarly lanes 5–8 contain 400, 300, 200, 100 ng of MtTOP respectively. B. Single-stranded DNA substrate PBAD (556 bases) amplified from pBAD/thio plasmid was utilized to map cleavage sites of EcTOP and MtTOP. Lanes 1–2: cleavage reactions containing 400, 300 ng of EcTOP respectively. Similarly lanes 3–4 contain 400, 300 ng of MtTOP respectively. Lanes A, G, C, T containing the sequencing reactions with the corresponding nucleotide termination mixes were electrophoresed along with EcTOP and MtTOP cleavage reactions to determine the cleavage sites.

Although the majority of topoisomerases do not have specific sequence requirements for cleavage sites, many of them show at least a certain degree of non -randomness in cleavage site recognition [6,20]. For example, EcTOP and *Micrococcal luteus *topoisomerase I cleave the sequence CXXX↓ (↓ represents the cleavage site) more preferentially than others [21]. Archeal and bacterial reverse gyrases, which are type IA topoisomerases, also have limited sequence requirements with only the preference of a cytosine or requirement of at least a pyrimidine at the -4 position of the cleavage site [22-24]. Previous studies elucidating the sequence specificity of topoisomerase I from *M. smegmatis *reported a strong topoisomerase I site (STS), wherein the enzyme recognizes and cleaves the sequence CG/TCT↓T [25,26]. We utilized different single-stranded 5'-^32^P labeled DNA substrates ranging from ~200–550 bases in length generated from either an *E. coli *plasmid or *M. tuberculosis *genomic DNA to characterize the MtTOP preferred cleavage sites. Results indicate that the DNA cleavage selectivity of MtTOP is very similar to that of EcTOP (Figure 6A, Table 1). The two enzymes share many cleavage sites on DNA derived either from *E. coli *or *M. tuberculosis*, but some cleavage sites were preferred by only one of these two enzymes (Figure 6B, Table 1). All of the cleavage sites for both enzymes were found to have a cytosine at the -4 position (CXXX↓) as previously shown for many bacterial topoisomerase I enzymes [21,27]. There was no specific cleavage sequence recognition for MtTOP as reported for *M. smegmatis *topoisomerase I.

**Table 1:** List of mapped DNA cleavage sites

Cleaved sequences^a^													Substrate
	-6	-5	-4	-3	-2	-1	1	2	3	4	5	6	
Sequences cleaved by both EcTOP and MtTOP													

Sequences preferred equally													
	G	G	C	A	T	T	T	C	G	G	T	G	MdnaK
	C	T	C	G	C	A	A	G	C	C	G	T	MdnaK
	T	T	C	C	A	A	C	G	G	A	T	C	MdnaK
	T	T	C	C	T	T	G	A	C	C	A	C	MdnaK
	T	G	C	C	A	T	A	T	T	T	C	G	PBAD
	T	T	C	A	A	G	C	T	T	G	T	C	PBAD
	C	T	C	G	T	C	T	T	C	C	A	T	Mtop
	T	T	C	C	A	T	C	G	C	C	G	C	Mtop
	G	A	C	C	G	A	A	A	T	G	C	T	Mtop
MtTOP preferred sequences^b^													
	T	T	C	C	A	G	T	C	G	G	T	G	MdnaK
	G	A	C	C	G	C	A	C	T	C	G	C	MdnaK
	C	A	C	G	T	T	G	T	G	C	T	C	MdnaK
	T	T	C	A	C	C	T	C	G	C	C	C	MdnaK
	A	T	C	G	T	C	A	T	C	A	C	C	PBAD
	T	T	C	A	A	C	T	G	A	C	C	T	PBAD
EcTOP preferred sequences^b^													
	A	G	C	C	C	T	T	C	C	A	G	T	MdnaK
	C	G	C	A	C	T	C	G	C	A	A	G	MdnaK
	A	T	C	A	C	G	T	T	G	T	G	C	MdnaK
	T	T	C	C	T	T	G	C	C	G	C	C	MdnaK
	G	T	C	A	T	C	A	C	C	G	G	A	PBAD

Sequences cleaved by MtTOP alone													

	G	T	C	G	T	C	G	C	C	G	C	C	MdnaK
	T	T	C	A	G	T	T	T	T	G	C	A	PBAD
	G	A	C	A	G	T	G	C	A	C	C	C	PBAD

Sequences cleaved by EcTOP alone													

	A	A	C	C	T	C	A	T	C	G	G	G	MdnaK
	C	G	C	C	G	A	G	G	T	G	G	T	MdnaK
	C	G	C	C	A	C	T	T	C	A	C	C	PBAD

^a ^Sequences are numbered from -6 to 6 (5' to 3'). Topoisomerase I mediated cleavage occurs between -1 and 1.

^b ^Among sequences cleaved by both EcTOP and MtTOP, sequences showing at least one-fold higher cleavage intensity towards EcTOP or MtTOP than the other enzyme are grouped as EcTOP or MtTOP preferred sequences respectively

## Discussion

Tuberculosis (TB) is the second leading cause of adult deaths due to infectious diseases world-wide, second only to HIV. The surge in multi-drug resistant *M. tuberculosis *makes it crucial to identify novel targets for development of new TB treatment. *M. tuberculosis *topoisomerase I could be one such novel target since there is only one type IA topoisomerase found in *M. tuberculosis*. A recent genome wide transposon mutagenesis experiment has postulated and categorised *M. tuberculosis topA *gene as essential [28]. It is also likely to be essential because every bacterium has at least one type IA topoisomerase activity. MtTOP is therefore an attractive target for drugs which would interfere with its relaxation activity (catalytic inhibitors). Moreover, besides inhibiting the overall relaxation activity of MtTOP, a more potent bactericidal effect could be achieved by drugs (catalytic poisons) that enhance the accumulation of covalent complexes on DNA, similar to the bactericidal mechanism of fluoroquinolones on type IIA bacterial topoisomerases. To aide such drug development efforts, it is important to have MtTOP protein in high purity and quantity. Here we report that by utilizing the *E. coli *Arctic express RP(DE3) strain, we took advantage of the higher GC rich codon usage efficiency and low temperature chaperone in this strain to obtain soluble MtTOP in high yield (12 mg from 7 L of bacterial culture). This enables future development of high through-put assays for inhibitors targeting MtTOP.

The DNA cleavage activity of MtTOP has not been characterized previously and there is a also a need for more detailed analysis of its DNA relaxation activity than in the early study of the enzyme [12]. Careful comparison with *E. coli *topoisomerase I (EcTOP) showed that the two enzymes had similar efficiency initially in relaxing the negatively supercoiled plasmid DNA isolated from *E. coli*. However, as the substrate plasmid DNA became partially relaxed, MtTOP was slower than EcTOP in removing the residual negative supercoils. This could be due to the different C-terminal domain found in the enzymes. The C-terminal domain found in EcTOP has been proposed to be important for substrate binding and coordination of strand passage during the relaxation cycle [29,30]. The C-terminal domain of MtTOP has no homology to the C-terminal domain in EcTOP, so it may function differently during the catalytic cycle. The N-terminal two-thirds, the transesterification domains of EcTOP and MtTOP have high degree of homology (41.9% identical).

Analysis of cleavage sites on both *E. coli *and *M. tuberculosis *derived DNA substrate showed that the cleavage site preferences are quite similar with a C in the -4 position as have been observed for several bacterial topoisomerase I as well as archeal and bacterial reverse gyrase enzymes. It is somewhat surprising that the cleavage site preference of MtTOP is not the same as that reported for *M. smegmatis *topoisomerase I (CG/TCT↓T). It is possible that this is due to the different experimental protocols used in analysis of the cleavage sites [25,26]. Besides *M. smegmatis *topoisomerase I, there are other examples of type IA topoisomerases that have cleavage site preferences different from that of EcTOP. These include CTT↓ for *E. coli *topoisomerase III [31], CANNN↓ for human topoisomerase III [32], ANN↓ for yeast topoisomerase III [33]. It remains unclear which part of the type IA enzyme structure determines the cleavage site selectivity. The specific sequence information for DNA cleavage by MtTOP should be useful in design of oligonucleotide substrates for DNA cleavage assays.

## Conclusion

A new procedure for expression and purification of recombinant MtTOP protein in high yield has been described. The enzyme is as efficient as EcTOP in initial removal of negative supercoils from plasmid DNA, but is less efficient than EcTOP in removing the remaining negative supercoils. The preferred DNA cleavage sites of MtTOP have limited sequence specificity but contain a C nucleotide in the -4 position, similar to most bacterial topoisomerase I and archeal reverse gyrase cleavage sites characterized previously.

## Methods

### MtTOP expression and purification

MtTOP was expressed from a recombinant plasmid pLIC-MTOP in *E.coli *Arctic express (DE3)RP strain (Stratagene). MtTOP coding sequence was amplified from the genomic DNA of *M. tuberculosis *H37RV strain with suitable primers (LIC-Mtop5'-TACTTCCAATCCAATGCAGCTGACCCGAAAACG and LIC-Mtop3'-TTATCCACTTCCAATGTTATTAGTCGCGCTTGGCTGC) using PfuUltra II Fusion HS DNA polymerase (Stratagene) and cloned into a vector pLIC-HK [34] through a ligation independent cloning procedure [35]. Cloning of MtTOP coding sequence into this vector containing a T7 promoter allowed T7 RNA polymerase dependent expression of MtTOP along with a tobacco etch virus (TEV) protease-cleavable N-terminal hexahistidine tag [34]. The resulting pLIC-MTOP plasmid, capable of expressing recombinant MtTOP was first isolated in *E. coli *NEB Turbo competent cells (New England Biolabs) and then transformed into Arctic express (DE3)RP cells after sequence confirmation. Expression of MtTOP in transformed Arctic express (DE3)RP cells was induced by 1 mM IPTG at 12°C according to the manufacturer's (Stratagene) protocol. After 24 h of induction, the cells were collected and subjected to freeze-thaw lysis [15] in lysis buffer (50 mM NaH_2_PO_4_, 300 mM NaCl, 10 mM imidazole, 1 mg/ml Lysozyme, pH 8.0). The recombinant protein in the soluble lysate was allowed to bind to Ni-NTA agarose (Qiagen) and packed into a column. After washing the column overnight with wash buffer (50 mM NaH_2_PO_4_, 300 mM NaCl, 20 mM imidazole, pH 8.0), the topoisomerase protein was eluted with an elution buffer (50 mM NaH_2_PO_4_, 300 mM NaCl, 250 mM Imidazole, pH 8.0) containing higher concentrations of imidazole. Eluted MtTOP was cleaved with TEV protease to remove the N-terminal hexa-histidine tag and purified by passing through a single-stranded DNA cellulose column as described [15].

### DNA Relaxation Activity assays

To assay for one unit of relaxation activity, EcTOP [16] and MtTOP enzymes of the same concentrations were diluted serially, ranging from 500–1 ng and assayed for DNA relaxation activity in a standard reaction volume of 20 μl with 10 mM Tris-HCl (pH 8.0), 50 mM NaCl, 0.1 mg/ml gelatin, 6 mM MgCl_2 _and 0.5 μg of supercoiled pBAD/thio plasmid DNA (purified by CsCl gradient centrifugation). After incubation at 37°C for 30 min, the reactions were stopped by adding 5 ul of 50 mM EDTA, 50% glycerol and 0.5%(v/v) bromophenol blue. The DNA was electrophoresed in a 1.0% (w/v) agarose gel with TAE buffer (40 mM Tris-acetate, pH 8.1, 2 mM EDTA). The gel was stained with ethidium bromide and photographed over UV light. One unit of enzyme was defined as the least quantity of the enzyme required for complete relaxation of negatively supercoiled DNA under the given reaction conditions.

Mg^2+ ^dependence of EcTOP and MtTOP to relax negatively supercoiled DNA was compared with either 50 ng or one unit of enzyme (100 ng of EcTOP, 500 ng of MtTOP) under varying concentrations of MgCl_2 _ranging from 0–20 mM over a time period of 30 min at 37°C with similar reaction conditions as described above. Also, a low concentration (50 ng) of EcTOP and MtTOP under standard conditions (6 mM MgCl_2_) as described earlier was used to compare the ability of the respective enzymes to relax negatively supercoiled DNA at various time points of 0, 10, 20, 30, 45, 60, 75, 90, 120 and 180 sec at 37°C.

### Cleavage of Single-stranded DNA

To compare and map the cleavage sites of EcTOP and MtTOP, single stranded DNA substrates were generated first by PCR (Table 2), followed by strand denaturation. Each of these substrates were radio-labeled at the 5' end by having one of the corresponding forward or reverse primers labeled with [γ-^32^P]ATP in the presence of T4 polynucleotide kinase prior to the PCR. The PCR products were purified using the DNA Clean and Concentrator Kit (Zymos) and eluted in TE buffer (10 mM Tris-HCl, pH 8.0, 1 mM EDTA). Prior to the addition of topoisomerase in the cleavage assay, the DNA substrate was denatured to single strands by heating at 95°C for 5 min and rapidly cooled on ice. After incubation with the topoisomerase at 37°C for 10 min, trapping of the covalent enzyme-DNA complex and cleaved DNA was achieved by the addition of 0.1 M NaOH. After neutralization, the DNA was electrophoresed in a 6% polyacrylamide sequencing gel followed by autoradiography of the dried gel to visualize the 5'-end-labeled DNA cleavage products. DNA sequencing reaction products were generated with the same 5' end labeled primer corresponding to that of the substrate used in the cleavage assay and by following the cycle sequencing procedures according to the manufacturer's instructions (SequiTherm DNA sequencing Kit, Epicentre). The sequencing reaction products were electrophoresed next to lanes containing cleavage products to identify the cleavage sites.

**Table 2:** Single stranded DNA substrates used

Substrate	Source	Primers	Substrate size (bases)	%GC
PBAD	pBAD/thio	Left-ATGCCATAGCATTTTTATCG Right-*GACCGGTACGCGTAGAATCG	556	47
MdnaK	*M. tuberculosis dnaK*	Left-*GAACCCGTTGTTCTTAGACGAG Right-*GGGTAACATCAAGCAGCAGAAC	317	62
Mtop	*M. tuberculosis topA*	Left-*GTAGAAGTTGTTGAGCCAGT Right-TACTCGTCGATCATCAAGAC	216	61

* Primer ^32^P labeled at 5'end to analyze extended strand as cleavage substrate after denaturation of the PCR product. The left and right primers of MdnaK were utilized in separate experiments.

## Authors' contributions

AA carried out the relaxation and DNA cleavage assays, and drafted the manuscript. ND and BC developed and carried out the expression and purification protocol. YT conceived the study, and participated in the design and coordination and helped to draft the manuscript. All authors read and approved the final manuscript.
